# A genomic biomarker signature can predict skin sensitizers using a cell-based *in vitro *alternative to animal tests

**DOI:** 10.1186/1471-2164-12-399

**Published:** 2011-08-08

**Authors:** Henrik Johansson, Malin Lindstedt, Ann-Sofie Albrekt, Carl AK Borrebaeck

**Affiliations:** 1 Department of Immunotechnology, Lund University, BMC D13, 21184 Lund, Sweden

## Abstract

**Background:**

Allergic contact dermatitis is an inflammatory skin disease that affects a significant proportion of the population. This disease is caused by an adverse immune response towards chemical haptens, and leads to a substantial economic burden for society. Current test of sensitizing chemicals rely on animal experimentation. New legislations on the registration and use of chemicals within pharmaceutical and cosmetic industries have stimulated significant research efforts to develop alternative, human cell-based assays for the prediction of sensitization. The aim is to replace animal experiments with in vitro tests displaying a higher predictive power.

**Results:**

We have developed a novel cell-based assay for the prediction of sensitizing chemicals. By analyzing the transcriptome of the human cell line MUTZ-3 after 24 h stimulation, using 20 different sensitizing chemicals, 20 non-sensitizing chemicals and vehicle controls, we have identified a biomarker signature of 200 genes with potent discriminatory ability. Using a Support Vector Machine for supervised classification, the prediction performance of the assay revealed an area under the ROC curve of 0.98. In addition, categorizing the chemicals according to the LLNA assay, this gene signature could also predict sensitizing potency. The identified markers are involved in biological pathways with immunological relevant functions, which can shed light on the process of human sensitization.

**Conclusions:**

A gene signature predicting sensitization, using a human cell line in vitro, has been identified. This simple and robust cell-based assay has the potential to completely replace or drastically reduce the utilization of test systems based on experimental animals. Being based on human biology, the assay is proposed to be more accurate for predicting sensitization in humans, than the traditional animal-based tests.

## Background

Allergic contact dermatitis (ACD) is a common inflammatory skin disease characterized by eczema and recurrent episodes of itching [1]. The disease affects a significant proportion of the population, with prevalence rates of 7.2% to 18.6% in Europe [2,3], and the incidence is increasing due to repeated exposure to sensitizing chemicals. ACD is a type IV delayed-type hypersensitivity response caused mainly by reactive T helper 1 (Th1) and interferon (IFN)γ producing CD8^+ ^T cells, at site of contact with small chemical haptens in previously exposed, and immunologically sensitized, individuals [4]. Dendritic cells (DC) in the epidermis initiate the immune reactions by responding to haptens bound to self-molecules subsequently activating T cell-mediated immunity.

The REACH (Registration, Evaluation, and Authorization of Chemicals) regulation requires that all new and existing chemicals within the European Union, involving approximately 30.000 chemicals, should be tested for hazardous effects [5]. As the identification of potential sensitizers currently requires animal testing, the REACH legislation will have a huge impact on the number of animals needed for testing. Further, the 7th Amendment to the Cosmetics Directive posed a ban on animal tests for the majority of cosmetic ingredients for human use, to be in effect by 2009, with the exceptions of some tests by 2013. Thus, development of reliable in vitro alternatives to experimental animals for the assessment of sensitizing capacity of chemicals is urgent. To date, no non-animal replacements are available for identification of skin sensitizing chemicals, instead the preferred assay is the mouse Local Lymph Node Assay (LLNA) [6], followed by the Guinea pig maximization test (GPMT) [7]. An *in vitro *alternative to these animal models should exhibit improved reliability, accuracy and importantly correlate to human reactivity.

DCs play key roles in the immune response by bridging the essential connections between innate and adaptive immunity. Upon stimulation, they can rapidly produce large amounts of mediators that affect chemotaxis and activation of other cells at the site of inflammation, and can selectively respond to various pathogens and environmental factors, by fine-tuning the cellular response through antigen-presentation. Thus, exploring and utilizing the immunological decision-making by DCs during stimulation with sensitizers, could serve as a potent test strategy for the prediction of sensitization.

Factors that complicate and impede the use of primary DCs as a test platform include adaptable phenotypes and specialized functions of different DC subpopulations, in addition to their wide and sparse distribution. Thus, the development of assays based on the predictability of DC function must rely on alternative cell types or mimics of *in vivo *DCs. For this purpose, a cell line with DC characteristics would be advantageous, as it constitutes a stable, reproducible and unlimited supply of cells. MUTZ-3 is an unlimited source of CD34^+ ^DC progenitors. Upon differentiation, MUTZ-3 can acquire phenotypes comparable to immature DCs or Langerhans-like DCs [8], present antigens through CD1d, MHC class I and II and induce specific T-cell proliferation [9]. Differentiated MUTZ-3 can also display a mature transcriptional and phenotypic profile upon stimulation with inflammatory cytokines [10].

In this report, we present a novel test principle for the prediction of skin sensitizers. To simplify the assay procedures and increase reproducibility, we employed progenitor MUTZ-3 cells, without further differentiation, and subjected the cells to stimulation with a large panel of sensitizing chemicals, non-sensitizing chemicals, and controls. The transcriptional response to chemical stimulation was assessed by genome-wide profiling. From data analysis, a biomarker signature of 200 transcripts was identified, which completely separated the response induced by sensitizing chemicals vs. non-sensitizing chemicals and the predictive power of the signature was illustrated, using ROC curves. The biomarker signature includes transcripts involved in relevant biological pathways, such as oxidative stress, DC maturation and cytokine responses, which further could shed light on molecular interactions involved in the process of sensitization. In conclusion, we have identified a biomarker signature with potent predictive power, which we propose as an in vitro assay for the identification of human sensitizing chemicals.

## Results

### The cellular rationale of the in vitro cell culture system

**Figure 1:**
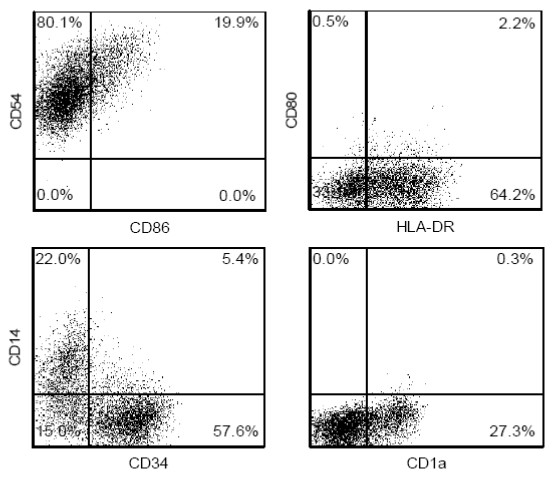
**Phenotype of MUTZ-3 cells prior to stimulation with sensitizing and non-sensitizing chemicals**. Cell surface expression levels of CD14, CD1a, CD34, CD54, CD80, CD86 and HLA-DR were assessed with flow cytometry. Gates were set to exclude debris and dead cells, and quadrants were established by comparing with relevant isotype controls. Results are shown from one representative experiment out of six.

DCs are essential immunoregulatory cells of the immune system demonstrated by their unique property to recognize antigen for the initiating of T cell responses, and their potent regulatory function in skewing immune responses. This makes them obvious targets for assay development. However, primary DCs constitute a heterogeneous and minor population of cells not suited for screening and the choice would be a human DC-like cell line, with characteristics compared to primary DCs. Since no leukemic cell line with DC-like properties has been reported [11], the generation of human DC-like cell lines relies on available myeloid leukemia cell lines. MUTZ-3 is a human acute myelomonocytic leukemia cell line with a potent ability to mimic primary human DCs [11]. Similar to immature primary DCs, MUTZ-3 progenitors express CD1a, HLA-DR and CD54, as well as low levels of CD80 and CD86 (Figure 1). The MUTZ-3 population also contains three subpopulations of CD14^+^, CD34^+ ^and double negative cells, previously reported to be transitional differentiation steps from a proliferative CD34^+ ^progenitor into a non-proliferative CD14^+ ^DC precursor [8]. Consequently, constitutively differentiating progenitor MUTZ-3 cells were used as the basis for a test system.

### CD86 surface expression in response to sensitizer stimulation

**Figure 2:**
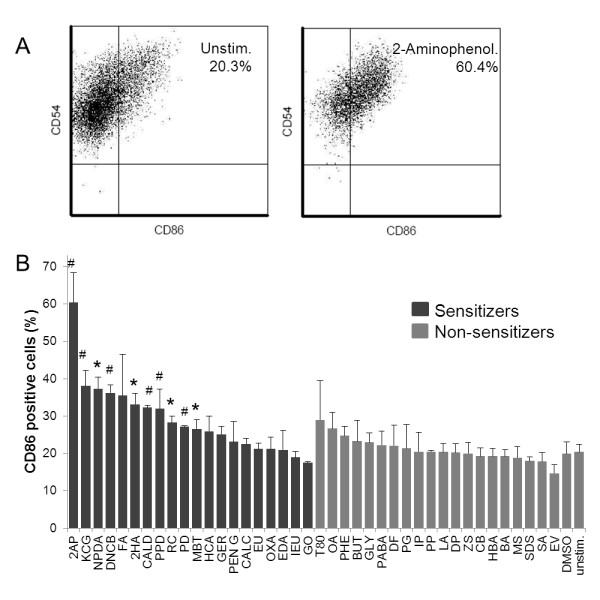
**Changes in CD86 expression following stimulation with sensitizing and non-sensitizing chemicals**. Cell surface expression levels of CD86 were monitored after stimulation with chemicals for 24 h. A). Chemical-induced up regulation of CD86, in terms of changes in frequency of positive cells, were determined by flow cytometry, as exemplified by the comparison of 2-aminophenol-stimulated cells (right dotplot) and unstimulated controls (left dot plot). Results are shown from one representative experiment out of three. Gates were set to exclude debris and dead cells, and quadrants were established by comparing with relevant isotype controls. B) Compilation of frequencies of CD86-positive cells after 24 h of stimulation. Statistical analysis was performed using Student's *t *test. *p < 0.05, # *p *< 0.01.

CD86 is the most extensively studied biomarker for sensitization to date, using e.g. monocyte derived dendritic cells (MoDCs) or human cell lines and their progenitors, such as THP-1, U-937 and KG-1. Thus, as a reference, cell surface expression of CD86 was measured with flow cytometry after 24 h stimulation, using 20 sensitizers and 20 non-sensitizers, as well as vehicle controls (Figure 2). CD86 was significantly up-regulated on cells stimulated with 2-aminophenol, kathon CG, 2-nitro-1,4-phenylendiamine, 2,4-dinitrochlorobenzene, 2-hydroxyethyl acrylate, cinnamic aldehyde, p-phenylendiamine, resorcinol, potassium dichromate, and 2-mercaptobenzothiazole. Hence, an assay based on measurement of a single biomarker, such as CD86, would give a sensitivity of 50% and a specificity of 100%. Consequently, CD86 cannot classify skin sensitizers, using a system based on MUTZ-3 cells.

### Analysis of the transcriptional profiles in chemically stimulated MUTZ-3 cells

**Figure 3:**
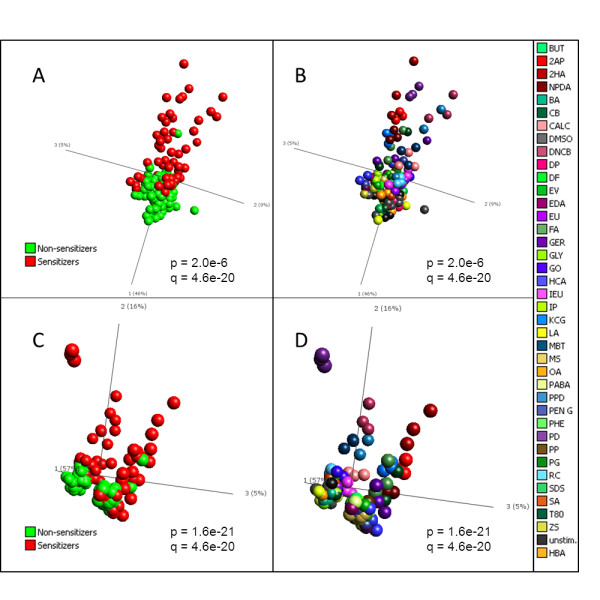
**Principal component analysis of transcripts differentially expressed after chemical stimulation**. mRNA levels in MUTZ-3 cells stimulated for 24 h with 20 sensitizing and 20 non-sensitizing chemicals were assessed with transcriptomics, using Affymetrix Human Gene 1.0 ST arrays. Structures and similarities in the gene expression data set were investigated, using principal component analysis (PCA) in the software Qlucore. A) PCA of genes differentially expressed in cells stimulated with sensitizing (red) versus non-sensitizing (green) chemicals (1010 genes identified with one-way ANOVA). B) PCA of genes differentially expressed in cells stimulated with sensitizing versus non-sensitizing chemicals (1010 genes), but now samples are colored by the compound used for stimulation. C) PCA of genes differentially expressed when comparing the different stimulations with 2-way ANOVA (1137 genes). Samples are colored according to sensitizing (red) and non-sensitizing (green) chemicals. D) PCA of genes differentially expressed when comparing the different stimulations with 2-way ANOVA (1137 genes), but now samples are colored by the compound used for stimulation. P, p-value from ANOVA. Q, p-value corrected for multiple hypothesis testing.

The genomic expression arrays were then used to test the same 20 sensitizers and 20 non-sensitizers, in triplicates. The vehicle controls, such as DMSO and distilled water, were included in twelve replicates. In total, a data set was generated based on 144 samples. RMA normalization and quality controls of the samples revealed that the oxazolone and cinnamic aldehyde samples were significant outliers and had to be removed, or they would have dominated the data set prohibiting biomarker identification (data not shown). In addition, one of the replicates of potassium permanganate had to be removed due to a faulty array. This left a data set consisting of 137 samples, each with data from measurements of 29,141 transcripts. In order to mine the data set for information specific for sensitizers vs. non-sensitizers, the software Qlucore Omics Explorer 2.1 was used, which enable real time principal component analysis (PCA) analysis. The input genes were at the same time sorted after desired criteria, i.e. sensitizers and non-sensitizers, based on ANOVA p-value selection. Two different ANOVA analyses were performed (Figure 3). First, Figure 3A and 3B show PCA plots based on 1010 transcripts with a p-value of ≤ 2.0 × 10^-6^, from a one-way ANOVA analysis, comparing sensitizing vs. non-sensitizing chemicals. As can be seen in Figure 3A, a clear discrimination can be made between the two groups, with non-sensitizers forming a condensed cloud in the lower part of the figure (green), while sensitizers stretch upwards in various directions (red). However, a complete separation is not achieved between the two groups at this level of significance. From Figure 3B, now colored according to stimulating agent, it is evident that one or more replicate of glyoxal, eugenol, hexylcinnamic aldehyde, isoeugenol, resorcinol, penicillin G and ethylendiamine grouped together with the control group. In addition, one replicate or more of the non-sensitizers tween 80, octanoic acid and phenol grouped closely with the sensitizers. Secondly, Figure 3C and 3D show PCA plots based on 1137 genes, with p-values ≤ 7.0 × 10^-21^, from a multi-group ANOVA analysis, comparing each individual stimulation. Identifying this large number of genes at this level of significance provided strong indications of the power in the data set. In Figure 3D, it is clear that the replicates group together, indicating high quality data. The triplicate samples of potassium dichromate have a discrete profile, which demonstrate a substantial impact of the cells compared to non-sensitizers. Furthermore, 2-hydroxyethyl acrylate, 2-aminophenol, kathon CG, formaldehyde, 2-nitro-1,4-phenylendiamine, 2,4-dinitrochlorobenzoic acid, p-phenylendiamine, 2-mercaptobenzothiazole, cinnamic alcohol and resorcinol have replicates that group together, separate from the negative group. Still, as can be seen in Figure 3C as well as in 3A, complete separation is not achieved with neither of the gene signatures of 1010 and 1137 genes both selected on p-values.

### Backward elimination identifies genes with the most discriminatory power

**Figure 4:**
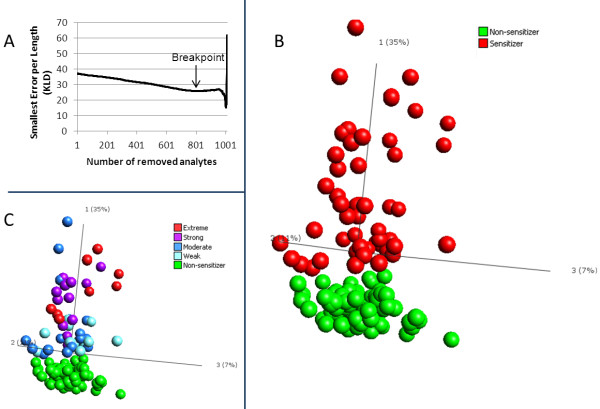
**Identification and PCA analysis of Prediction Signature**. A) The number of differentially expressed significant genes in cells stimulated with sensitizing versus non-sensitizing chemicals (1010 genes) was reduced, using Backward Elimination. The lowest KLD is observed after elimination of 810 analytes, referred to as the Breakpoint. The remaining 200 genes are considered to be the top predictors in the data set, and are termed Prediction Signature. B) Complete separation between sensitizers (red) and non-sensitizers (green) is observed with PCA of the Prediction Signature. C) Same PCA as in B, now with samples colored according to their potency in LLNA.

**Figure 5:**
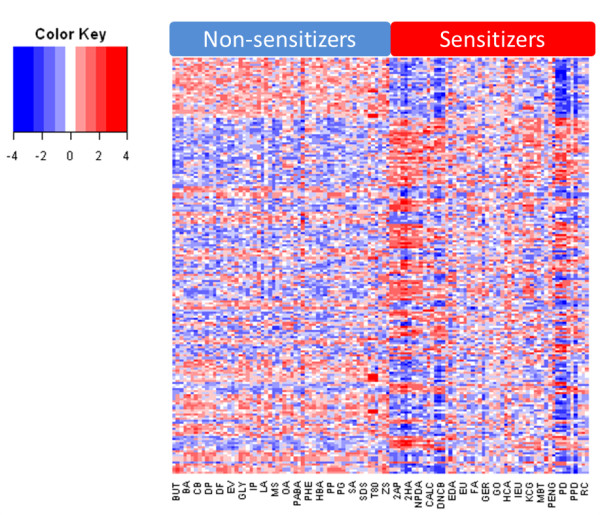
**Transcriptional profiles of sensitizers and non-sensitizers**. Hierarchical clustering of the genes in the Prediction Signature. Samples are grouped as sensitizer or non-sensitizer, and all replicates are included. Each row represents one gene, which is scaled to have a mean of zero and standard deviation of one, with colors representing the number of standard deviations from the mean.

Even though the data set contains genes with p-values down to 1 × 10^-17^, lowering the p-value cutoff did not achieve complete separation between sensitizers and non-sensitizers. Gene signatures entirely selected on p-values does not provide the best possible predictive power, since the information is *per se *not orthogonal. To further reduce the number of transcripts for a predictive biomarker signature, we employed an algorithm for backward elimination (Figure 4A). The algorithm removes genes one by one while taking into account not only the impact of genes individually, but how they perform collectively with the entire selected gene signature. For each gene eliminated, the Kullback-Leibler divergence (KLD) value is lowered, until a breakpoint is reached, at which point 200 genes remained. Continuing eliminating genes at this point causes the KLD to rise again, indicating that information is being lost (Figure 4A). Therefore, the 200 genes with lowest KLD value were selected for further analysis. PCA of the 200 analytes now revealed that they have the ability to completely separate sensitizers from non-sensitizers, indicating that these transcripts can be used as predictors for sensitizing properties of unknown samples (Figure 4B). Importantly, by coloring the samples in the PCA by their potency, according to LLNA, it is clear that potency can also be predicted (Figure 4C), as extreme and strong sensitizers tend to group further from the non-sensitizers, while moderate and extreme sensitizers group closer to non-sensitizers. The 200 genes are termed the "Prediction Signature" and their identities are listed in Table 1. In addition, the transcriptional profiles of the differentially expressed genes are presented in a heatmap (Figure 5).

**Table 1:** Prediction Signature

Gene Title	Gene Symbol	Entrez Gene ID	Affymetrix HuGene 1.0 ST ID	Validation Call frequency (%)
4-aminobutyrate aminotransferase	ABAT	18	7993126	30
abhydrolase domain containing 5	ABHD5	51099	8079153	85
alkaline ceramidase 2	ACER2	340485	8154563	95
ATP citrate lyase	ACLY	47	8015460	85
actin-related protein 10 homolog (S. cerevisiae)	ACTR10	55860	7974587	75
ADAM metallopeptidase domain 20	ADAM20	8748	7979927	35
aldehyde dehydrogenase 18 fam., member A1	ALDH18A1	5832	7935230	75
aldehyde dehydrogenase 1 fam., member B1	ALDH1B1	219	8155327	70
anaphase promoting complex subunit 1	ANAPC1	64682	8043349	55
anaphase promoting complex subunit 5	ANAPC5	51433	7967149	25
ankyrin repeat, fam. A (RFXANK-like), 2	ANKRA2	57763	8112596	100
ADP-ribosylation factor GTPase activating protein 3	ARFGAP3	26286	8076515	55
Rho GTPase activating protein 9	ARHGAP9	64333	7964436	75
ankyrin repeat and SOCS box-containing 7	ASB7	140460	7986433	65
ATPase, H+ transporting, lysosomal 38 kDa, V0 subunit d1//ATPase, H+ transporting, lysosomal 38 kDa, V0 subunit d1	ATP6V0D1//ATP6V0D1	9114//9114	8002041	10
ATPase, H+ transporting, lysosomal 9 kDa, V0 subunit e1	ATP6V0E1	8992	8110022	75
ATPase, H+ transporting, lysosomal 50/57 kDa, V1 subunit H	ATP6V1H	51606	8150797	100
B-cell CLL/lymphoma 7A	BCL7A	605	7959354	85
bridging integrator 2	BIN2	51411	7963289	80
bleomycin hydrolase	BLMH	642	8014008	15
brix domain containing 1//ribosome production factor 2 homolog (S. cerevisiae)	BXDC1//RPF2	84154//84154	8062211	40
chromosome 11 open reading frame 61	C11orf61	79684	7952445	55
chromosome 11 open reading frame 67//integrator complex subunit 4	C11orf67//INTS4	28971//92105	7942783	50
chromosome 12 open reading frame 57	C12orf57	113246	7953564	40
chromosome 13 open reading frame 18	C13orf18	80183	7971486	50
chromosome 15 open reading frame 24	C15orf24	56851	7987172	50
chromosome 19 open reading frame 46//alkB, alkylation repair homolog 6 (E. coli)	C19orf46//ALKBH6	163183//84964	8036242	30
chromosome 19 open reading frame 54	C19orf54	284325	8036956	95
chromosome 1 open reading frame 174	C1orf174	339448	7911897	40
chromosome 1 open reading frame 183	C1orf183	55924	7918552	85
chromosome 20 open reading frame 111	C20orf111	51526	8066402	65
chromosome 20 open reading frame 24	C20orf24	55969	8062326	20
chromosome 3 open reading frame 62//ubiquitin specific peptidase 4 (proto-oncogene)	C3orf62//USP4	375341//7375	8087374	40
chromosome 9 open reading frame 89	C9orf89	84270	8156404	100
coactivator-associated arginine methyltransferase 1	CARM1	10498	8025766	60
CD33 molecule	CD33	945	8030804	45
CD86 molecule	CD86	942	8082035	45
CD93 molecule	CD93	22918	8065359	50
cytochrome c oxidase subunit VIIa polypeptide 2 like	COX7A2L	9167	8051777	45
corticotropin releasing hormone binding protein	CRHBP	1393	8106418	45
chondroitin sulfate N-acetylgalactosaminyltransferase 2	CSGALNACT2	55454	7927146	90
cytochrome P450, fam. 51, subfam. A, polypeptide 1	CYP51A1	1595	8140864	85
DDRGK domain containing 1	DDRGK1	65992	8064601	60
DEAD (Asp-Glu-Ala-As) box polypeptide 19A	DDX19A	55308	7997059	95
DEAD (Asp-Glu-Ala-Asp) box polypeptide 21	DDX21	9188	7927936	60
24-dehydrocholesterol reductase	DHCR24	1718	7916432	100
7-dehydrocholesterol reductase	DHCR7	1717	7950067	80
DEAH (Asp-Glu-Ala-His) box polypeptide 33	DHX33	56919	8011861	100
DnaJ (Hsp40) homolog, subfam. B, member 4	DNAJB4	11080	7902512	100
DnaJ (Hsp40) homolog, subfam. B, member 9	DNAJB9	4189	8135480	25
DnaJ (Hsp40) homolog, subfam. C, member 5	DNAJC5	80331	8064208	10
DnaJ (Hsp40) homolog, subfam. C, member 9	DNAJC9	23234	7934320	55
DNA-damage regulated autophagy modulator 2//choline/ethanolamine phosphotransferase 1	DRAM2//CEPT1	128338//10390	7918474	100
D-tyrosyl-tRNA deacylase 1 homolog (S. cerevisiae)	DTD1	92675	8061211	45
ER degradation enhancer, mannosidase alpha-like 2	EDEM2	55741	8065855	80
ecotropic viral integration site 2B	EVI2B	2124	8014063	60
fam. with sequence similarity 36, member A//non-protein coding RNA 201	FAM36A//NCRNA00201	116228//284702	7911085	15
fam. with sequence similarity 86, member A	FAM86A	196483	7999304	25
Fas (TNF receptor superfam., member 6)	FAS	355	7929032	70
fatty acid synthase	FASN	2194	8019392	100
F-box protein 10//translocase of outer mitochondrial membrane 5 homolog (yeast)	FBXO10//TOMM5	26267//401505	8161229	40
MGC44478	FDPSL2A	619190	8140443	55
ferredoxin reductase	FDXR	2232	8018236	40
forkhead box O4	FOXO4	4303	8168205	80
ferritin, heavy polypeptide-like 5	FTHL5	2509	8126948	95
fucosidase, alpha-L- 2, plasma	FUCA2	2519	8129974	20
growth arrest-specific 2 like 3	GAS2L3	283431	7957850	70
ganglioside induced differentiation associated protein 2	GDAP2	54834	7918955	80
growth differentiation factor 11	GDF11	10220	7956026	65
glutaredoxin (thioltransferase)	GLRX	2745	8113214	90
guanine nucleotide binding protein-like 3 (nucleolar)-like	GNL3L	54552	8167797	85
glucosamine-phosphate N-acetyltransferase 1	GNPNAT1	64841	7979196	90
glutathione reductase	GSR	2936	8150112	40
GTF2I repeat domain containing 2//GTF2I repeat domain containing 2B	GTF2IRD2//GTF2IRD2B	84163//389524	8133549 and 8140170	50 and 30
general transcription factor IIIC, polypeptide 2, beta 110 kDa	GTF3C2	2976	8051075	55
HMG-box transcription factor 1//component of oligomeric golgi complex 5	HBP1//COG5	26959//10466	8135392	65
histone cluster 1, H1c	HIST1H1C	3006	8124397	45
histone cluster 1, H1e	HIST1H1E	3008	8117377	95
histone cluster 1, H2ae	HIST1H2AE	3012	8117408	45
histone cluster 1, H2be	HIST1H2BE	8344	8117389	15
histone cluster 1, H3g	HIST1H3G	8355	8124440	35
histone cluster 1, H3j	HIST1H3J	8356	8124537	60
histone cluster 1, H4a	HIST1H4A	8359	8117334	10
histone cluster 2, H2ac//histone cluster 2, H2aa3//histone cluster 2, H2aa4	HIST-2H2AC//2H2AA3//2H2AA4	8338//8337//723790	7905079 and 7919619	75 and 75
histone cluster 2, H2bf//histone cluster 2, H2be//histone cluster 2, H2ba	HIST-2H2BF//2H2BE//2H2BA	440689//8349//337875	7919606	50
high-mobility group box 3	HMGB3	3149	8170468	5
3-hydroxy-3-methylglutaryl-Coenzyme A reductase//3-hydroxy-3-methylglutaryl-CoA reductase	HMGCR//HMGCR	3156//3156	8106280	90
3-hydroxy-3-methylglutaryl-Coenzyme A synthase 1 (soluble)//3-hydroxy-3-methylglutaryl-CoA synthase 1 (soluble)	HMGCS1//HMGCS1	3157//3157	8111941	80
heme oxygenase (decycling) 1	HMOX1	3162	8072678	10
heterogeneous nuclear ribonucleoprotein L	HNRNPL	3191	8036613	30
insulin receptor substrate 2	IRS2	8660	7972745	35
iron-sulfur cluster scaffold homolog (E. coli)	ISCU	23479	7958414	100
interferon stimulated exonuclease gene 20 kDa-like 2	ISG20L2	81875	7921110	45
potassium voltage-gated channel, Isk-related fam., member 3	KCNE3	10008	7950409	25
keratinocyte growth factor-like protein 1//fibroblast growth factor 7 (keratinocyte growth factor)//keratinocyte growth factor-like protein 2//hypothetical protein FLJ20444	KGFLP1//FGF7//KGFLP2//FLJ20444	387628//2252//654466//403323	8155530	70
lysophosphatidic acid receptor 1	LPAR1	1902	8163257	10
leucine-rich PPR-motif containing	LRPPRC	10128	8051882	65
lymphocyte antigen 96	LY96	23643	8146934	35
mitogen-activated protein kinase kinase 1//small nuclear RNA activating complex, polypeptide 5, 19 kDa	MAP2K1//SNAPC5	5604//10302	7984319	30
mitogen-activated protein kinase 13	MAPK13	5603	8119016	60
methyltransferase like 2A	METTL2A	339175	8009008	45
microsomal glutathione S-transferase 3	MGST3	4259	7906978	70
mitochondrial ribosomal protein L30	MRPL30	51263	8043848	30
mitochondrial ribosomal protein L4	MRPL4	51073	8025586	40
mitochondrial ribosomal protein S17//glioblastoma amplified sequence//zinc finger protein 713	MRPS17//GBAS//ZNF713	51373//2631//349075	8132922	60
mitochondrial poly(A) polymerase//golgi autoantigen, golgin subfam. a, 6 pseudogene	MTPAP//LOC729668	55149//729668	7932834	45
5-methyltetrahydrofolate-homocysteine methyltransferase	MTR	4548	7910752	15
neighbor of BRCA1 gene 1	NBR1	4077	8007471	20
nuclear import 7 homolog (S. cerevisiae)	NIP7	51388	7996934	75
NLR fam., pyrin domain containing 12	NLRP12	91662	8039096	35
nucleolar protein fam. 6 (RNA-associated)	NOL6	65083	8160682	95
NAD(P)H dehydrogenase, quinone 1	NQO1	1728	8002303	45
nuclear receptor binding protein 1	NRBP1	29959	8040927	20
nucleotide binding protein-like	NUBPL	80224	7973826	10
nudix (nucleoside diphosphate linked moiety X)-type motif 14	NUDT14	256281	7981566	35
nuclear fragile × mental retardation protein interacting protein 1	NUFIP1	26747	7971361	60
nucleoporin 153 kDa	NUP153	9972	8124059	25
olfactory receptor, fam. 5, subfam. B, member 21	OR5B21	219968	7948330	50
PAS domain containing serine/threonine kinase	PASK	23178	8060205	55
PRKC, apoptosis, WT1, regulator	PAWR	5074	7965112	30
PDGFA associated protein 1	PDAP1	11333	8141273	35
phosphodiesterase 1B, calmodulin-dependent	PDE1B	5153	7955943	85
phosphoribosylformylglycinamidine synthase	PFAS	5198	8004804	60
pleckstrin homology-like domain, fam. A, member 3	PHLDA3	23612	7923372	75
phosphoinositide-3-kinase adaptor protein 1	PIK3AP1	118788	7935337	20
PTEN induced putative kinase 1	PINK1	65018	7898663	70
phosphomannomutase 2	PMM2	5373	7993148	65
partner of NOB1 homolog (S. cerevisiae)	PNO1	56902	8042381	40
polymerase (RNA) II (DNA directed) polypeptide E, 25 kDa	POLR2E	5434	8032149	80
polymerase (RNA) III (DNA directed) polypeptide E (80 kD)	POLR3E	55718	7993973	30
protein phosphatase 1D magnesium-dependent, delta isoform//protein phosphatase, Mg2+/Mn2+ dependent, 1D	PPM1D//PPM1D	8493//8493	8008922	80
phosphatidylinositol-3,4,5-trisphosphate-dependent Rac exchange factor 1	PREX1	57580	8066848	100
proline-serine-threonine phosphatase interacting protein 1	PSTPIP1	9051	7985099	95
prothymosin, alpha	PTMA	5757	7954006 and 7961022	20 and 15
RAB33B, member RAS oncogene fam.	RAB33B	83452	8097507	40
renin binding protein	RENBP	5973	8175933	65
replication factor C (activator 1) 2, 40 kDa	RFC2	5982	8140151	30
ribonuclease H1	RNASEH1	246243	8050079	90
ring finger protein 146	RNF146	81847	8121927	50
ring finger protein 24	RNF24	11237	8064766	100
ring finger protein 26	RNF26	79102	7944510	95
ribosomal protein SA//small nucleolar RNA, H/ACA box 62	RPSA//SNORA62	3921//6044	8078918	75
RNA pseudouridylate synthase domain containing 2	RPUSD2	27079	7982753	45
ribosomal RNA processing 12 homolog (S. cerevisiae)	RRP12	23223	7935425	75
retinoid × receptor, alpha	RXRA	6256	8159127	5
scavenger receptor class B, member 2	SCARB2	950	8101158	70
SERPINE1 mRNA binding protein 1	SERBP1	26135	7916836	95
splicing factor proline/glutamine-rich (polypyrimidine tract binding protein associated)//splicing factor proline/glutamine-rich	SFPQ//SFPQ	6421//6421	7914791	40
solute carrier fam. 25, member 32//DDB1 and CUL4 associated factor 13	SLC25A32//DCAF13	81034//25879	8152255	100
solute carrier fam. 35, member B3	SLC35B3	51000	8123825	40
solute carrier fam. 37 (glucose-6-phosphate transporter), member 4	SLC37A4	2542	7952132	55
solute carrier fam. 5 (sodium-dependent vitamin transporter), member 6	SLC5A6	8884	8051030	95
sphingomyelin phosphodiesterase 4, neutral membrane (neutral sphingomyelinase-3)	SMPD4	55627	8055183	40
small nucleolar RNA host gene 1 (non-protein coding)//small nucleolar RNA, C/D box 26	SNHG1//SNORD26	23642//9302	7948908	20
small nucleolar RNA host gene 12 (non-protein coding)	SNHG12	85028	7914202	10
small nucleolar RNA, H/ACA box 45	SNORA45	677826	7938293	25
sorting nexin fam. member 27	SNX27	81609	7905444	35
spinster homolog 2 (Drosophila)//MYB binding protein (P160) 1a	SPNS2//MYBBP1A	124976//10514	8011640	45
sprouty homolog 2 (Drosophila)	SPRY2	10253	7972217	75
squalene epoxidase	SQLE	6713	8148280	95
sterol regulatory element binding transcription factor 2	SREBF2	6721	8073522	45
ST3 beta-galactoside alpha-2,3-sialyltransferase 6	ST3GAL6	10402	8081219	100
serine/threonine kinase 17b	STK17B	9262	8057887	90
transmembrane anterior posterior transformation 1	TAPT1	202018	8099506	65
taste receptor, type 2, member 5	TAS2R5	54429	8136647	40
tubulin folding cofactor E-like	TBCEL	219899	7944623	55
tectonic fam. member 2	TCTN2	79867	7959638	40
toll-like receptor 6	TLR6	10333	8099841	30
transmembrane protein 150B	TMEM150B	284417	8039453	25
transmembrane protein 55A	TMEM55A	55529	8151756	90
transmembrane protein 59	TMEM59	9528	7916372	90
transmembrane protein 97	TMEM97	27346	8005839	95
tumor necrosis factor receptor superfam., member 10c, decoy without an intracellular domain	TNFRSF10C	8794	8145244	75
translocase of outer mitochondrial membrane 34	TOMM34	10953	8066461	35
translocase of outer mitochondrial membrane 40 homolog (yeast)	TOMM40	10452	8029521	40
tumor protein p53 inducible protein 3	TP53I3	9540	8050702	30
tumor protein p53 inducible nuclear protein 1	TP53INP1	94241	8151890	100
twinfilin, actin-binding protein, homolog 2 (Drosophila)//toll-like receptor 9	TWF2//TLR9	11344//54106	8087860	65
thioredoxin reductase 1	TXNRD1	7296	7958174	55
ubiquitin-fold modifier conjugating enzyme 1	UFC1	51506	7906662	95
ubiquitin specific peptidase 10	USP10	9100	7997633	30
vesicle-associated membrane protein 3 (cellubrevin)	VAMP3	9341	7897370	40
valyl-tRNA synthetase	VARS	7407	8125091 and 8178609	10 and 10
vacuolar protein sorting 37 homolog A (S. cerevisiae)	VPS37A	137492	8144774	60
zinc finger protein 211	ZNF211	10520	8031792	45
zinc finger protein 223	ZNF223	7766	8029360	65
zinc finger protein 561	ZNF561	93134	8033795	60
zinc finger protein 79	ZNF79	7633	8158022	100
---	---	---	7910385	40
---	---	---	7946567	15
---	---	---	7966223	45
---	---	---	7979694	40
---	---	---	8130495	30
---	---	---	8180237	60
---	---	---	8180268	85
---	---	---	8180417	85

The table shows the biomarker genes found by t-test and Backward Elimination. Genes were annotated, using the NetAffx database from Affymetrix (http://www.affymetrix.com, Santa Clara USA). When found, the Entrez Gene ID http://www.ncbi.nlm.nih.gov/gene was chosen as the gene identifier. The validation call frequency (%) is the occurrence of each gene in the 20 Test Gene Signatures obtained in the validation step.

### Interrogation of the analysis used to identify the Prediction Signature

**Figure 6:**
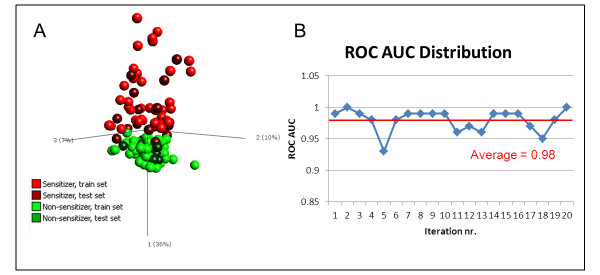
**Validation of selection procedure of Prediction Signature**. The method by which the Prediction Signature was constructed was validated by repeating the process on 70% randomly selected data (training set). The remaining 30% of data was used as a test set for signature validation. The process was repeated for 20 iterations. A) A representative PCA of one of the 20 iterations, which demonstrates that the Test Gene Signature can separate skin sensitizers from non-sensitizers. Only the samples of the 70% training set, displayed in bright colors, were used to build the space of the first three principal components. The test set samples, displayed in dark colors, were plotted into this space based on expression levels of the analytes in the Test Gene Signature. B) An SVM was trained on the 70% training set, and validated with the 30% test set. The areas under the ROC curve from 20 such randomizations are plotted, yielding an average AUC value of 0.98. This indicated that the classification of samples in the test set was correct.

To validate the predictive power of our signature, we used a machine learning method called the Support Vector Machine (SVM) [12], which maps the data from a training set in space in order to maximize the separation of gene expression induced by sensitizing and non-sensitizing chemicals. As training set, 70% of the data set was selected randomly and the entire selection process was repeated. Starting with 29,141 transcripts, the signature was reduced to 200 transcripts, termed "Test Gene Signature", using ANOVA filtering and backward elimination, as described above. The remaining 30% of the data set was used to test each signature. The partitioning of the data set into subsets of 70% training data set and 30% test data set was done in a stratified random manner, while maintaining the relation of sensitizers and non-sensitizers. Thereafter, the Test Gene Signature was used to train an SVM model with the training set, and the predictive power of the model was assessed with the test set. This entire process was iterated 20 times. The frequency by which each gene in the Prediction Signature was included in the Test Gene Signatures is reported in table 1. Figure 6A shows a PCA plot based on the Test Gene Signature from one representative iteration. Clearly, the separation between sensitizers and non-sensitizers resembles the one observed for the Prediction Signature in Figure 4B. In Figure 6A, the samples of the sensitizing and non-sensitizing chemicals in the test set have been colored dark red and dark green respectively, indicating that they are not contributing to the principal components of the plot, but are merely plotted based on their expression values of the selected Test Gene Signature. As can be seen, sensitizers from the test set group with sensitizers from the training set, while non-sensitizers from the test set group with non-sensitizers from the training set. The final outcome of the SVM training and validation can be seen in Figure 6B, where the areas under the ROC curve are plotted for each iteration. The average area under the ROC curve of 0.98 confirms the ability to discriminate sensitizers from control samples. Based on this average, the estimated prediction performance of the assay reveals an accuracy of 99%, sensitivity of 99% and specificity of 99%. While this experiment does not validate the prediction power of the Prediction Signature *per se*, it does indeed validate the method by which it has been selected, supporting the claim that the Prediction Signature is capable of accurately predicting sensitizing properties of unknown samples.

### Interactome, molecular functions and canonical pathways involving the Prediction Signature

Using Ingenuity Pathways Analysis (IPA, Ingenuity Systems Inc.), 184 of the 200 molecules in the signature were characterized with regard to the interactome, known functions and (canonical) pathways. The remaining 16 molecules could not be mapped to any unique IPA entries. The dominating functions identified were small molecule biochemistry (39 molecules), cell death (33), lipid metabolism (25), hematological system development (18), cell cycle (18), molecular transport (17), cellular growth and proliferation (16), and carbohydrate metabolism (15) (Table 2).

**Table 2:** Dominating functions of the Prediction signature

Function	Number of molecules from signature	Molecule names	Most prominent sub functions
small molecule biochemistry	39	ABHD5, ACLY, ALDH18A1, BLMH, CD86, CSGALNACT2, CYP51A1, DHCR24, DHCR7, DNAJC5, FAS, FASN, FDXR, FOXO4, GLRX, GNPNAT1, HMGCR, HMOX1, IRS2, LPAR1, LY96, MGST3, MTR, NQO1, PASK, PDE1B, PINK1, PMM2, RENBP, RXRA, SLC25A32, SLC37A4, SLC5A6, SMPD4, SQLE, SREBF2, ST3GAL6, TLR6, TMEM55A	Metabolism (24), biosynthesis (15), modification (12), synthesis (11)

cell death	33	CD33, DDX19A, DHCR24, DNAJB9, DNAJC5, FAS, FASN, FDXR, FOXO4, GLRX, GNPNAT1, GSR, HIST1H1C, HMGB3, HMOX1, IRS2, LPAR1, MAP2K1, MAPK13, NQO1, PAWR, PDE1B, PHLDA3, PINK1, PPM1D, RXRA, SERBP1, SPRY2, STK17B, TLR6, TNFRSF10C, TP53INP1, TXNRD1	Apoptosis (30), cell death (13)

lipid metabolism	25	ABHD5, ACLY, CYP51A1, DHCR24, DHCR7, FAS, FASN, FDXR, FOXO4, HMGCR, HMOX1, IRS2, LPAR1, LY96, MGST3, PASK, RENBP, RXRA, SLC37A4, SMPD4, SQLE, SREBF2, ST3GAL6, TLR6, TMEM55A	Metabolism (18), synthesis (11), modification (11)

hematological system development	18	CARM1, CD33, CD86, FAS, FOXO4, HMGB3, HMGCR, HMOX1, IRS2, LY96, NBR1, NQO1, PAWR, PIK3AP1, PPM1D, STK17B, TP53INP1, VAMP3	Proliferation (10), quantity (7)

cell cycle	18	ABHD5, ANAPC5, DNAJB4, DTD1, FAS, FASN, FOXO4, GDF11, HBP1, HMOX1, IRS2, MAP2K1, PAWR, PPM1D, RXRA, SFPQ, SPRY2, TP53INP1	Cell cycle progression (13), G2 phase (5)

molecular transport	17	ABHD5, DNAJC5, FAS, FOXO4, HMOX1, LPAR1, MTR, NQO1, PASK, PINK1, RENBP, RXRA, SLC25A32, SLC37A4, SLC5A6, SREBF2, TLR6	Accumulation (9), quantity (5)

cellular growth and proliferation	16	CD33, CD86, FAS, GNPNAT1, HMOX1, IRS2, LPAR1, LY96, MAP2K1, PAWR, PIK3AP1, PPM1D, RXRA, SPRY2, STK17B, TP53INP1	Proliferation (16), growth (4)

carbohydrate metabolism	15	ABHD5, ACLY, CSGALNACT2, FAS, FASN, FUCA2, GNPNAT1, IRS2, LY96, NQO1, PMM2, RENBP, SLC37A4, ST3GAL6, TMEM55A	Metabolism (9), biosynthesis (5)

Dominating functions in the molecular signature. 184 of the 200 molecules were functionally investigated, using IPA. Only functions populated by 15 or more genes were included in the present study.

Pathways possibly invoked by the molecules in the signature were also investigated using IPA. Those most highly populated involved NRF2-mediated oxidative response (10), xenobiotic metabolism signaling (8), protein ubiquitination pathway (7), LPS/IL-1 mediated inhibition of RXR function (6), aryl hydrocarbon receptor signaling (6) and protein kinase A signaling (6). These pathways are known to take part in reactions provoked by foreign substances, xenobiotics, which supports a relevant biology behind the genomic signature.

## Discussion

Allergic contact dermatitis (ACD) is an inflammatory skin disease caused by an adaptive immune response to normally innocuous chemicals [13]. Small molecular weight chemicals, so-called haptens, can bind self-proteins in the skin, which enables internalization of the protein-bound allergenic chemical by skin dendritic cell (DC). DCs, under the influence of the local microenvironment, process the protein-hapten complex, migrate to the local lymph nodes and activate naïve T cells. The initiation and development of allergen-specific responses, mainly effector CD8+ T cells and Th1 cells, and production of immunoregulatory proteins, are hallmarks of the immune activation observed in ACD. ACD is also the most common manifestation of immunotoxicity observed in humans [13] and hundreds of chemicals have been shown to cause sensitization in skin [14]. The driving factors and molecular mechanisms involved in sensitization are still unknown even though intense research efforts have been carried out to characterize the immunological responses towards allergenic chemicals. The REACH legislation requires that all chemicals produced over 1 ton/year are tested for hazardous properties such as toxicity and allergenicity [5], which increase the demand for accurate assays with predictive power for hazard identification. Additionally, the 7th Amendment to the Cosmetics Directive (76/768/EEC) poses a complete ban on using animal experimentation for testing cosmetic ingredients by 2013 if a scientifically reliable method is available. Thus, there is a significant need for predictive test methods that are based on human cells. Today, the identification of potential human sensitizers relies on animal experimentation, in particular the murine local lymph node assay (LLNA) [6]. The LLNA is based upon measurements of proliferation induced in draining lymph nodes of mice after chemical exposure [15]. Chemicals are defined as sensitizers if they provoke a three-fold increase in proliferation compared to control, and the amount of chemical required for the increase is the EC3 value. Thus, the LLNA can also be used to categorize the chemicals based on sensitization potency. However, LLNA is, besides the obvious ethical implications, also time consuming and expensive. Human sensitization data often stem from human maximization tests (HMT) [16] and human patch tests (HPT). In an extensive report from the Interagency Coordinating Committee on the Validation of Alternative Methods (ICCVAM), the performance characteristics of LLNA were compared to other available animal-based methods and human sensitization data (HMT and HPT) [17]. The LLNA performance in comparison to human data (74 assessments) revealed an accuracy of 72%, a sensitivity of 72% and a specificity of 67%.

Various human cell lines and primary cells involved in sensitization have been evaluated as predictive test system, such as epithelial cells, dendritic cells and T cells, however, no validated test assay is currently available. THP-1, U937, KG-1 and MUTZ-3, naive or differentiated, are among the human myeloid cell lines most extensively evaluated as platforms for DC-based in vitro assays, as reviewed in [18]. These cells are easy to grow and enable standardization of protocols. U937 and THP-1 are currently being evaluated in pre-validation stage for prediction of skin sensitization. The Human Cell Line Activation Test (hCLAT) is based upon analysis of CD86 and/or CD54 expression on THP-1 cells after chemical stimulation [19,20]. The Myeloid U937 Skin Sensitization Test (MUSST) also involves analysis of CD86 [21]. These assays are thus very limited in readout. As CD86 is among the markers most extensively studied, we evaluated the expression level of this marker in our assay. We demonstrated its relevance but also its insufficient predictive power (Figure 2), since only 10 out of 20 sensitizing chemicals induced a significant up regulation of CD86. Various other single biomarkers have been suggested to be up regulated upon stimulation with sensitizing chemicals, such as CD40, CD80, CD54, CXCL8, IL-1β, MIP-1β, p38 MAPK, as reviewed in [18], yet single-handedly, none of them have enough predictive power to discriminate between sensitizing and non-sensitizing chemicals. The analysis of biomarker signatures, i.e. combination of biomarkers, has been shown to be superior in molecular diagnostic of cancer and superior to any single biomarker. Consequently, we therefore utilized the power of global transcriptomics and screened the gene regulation induced by a large set of well-defined chemicals and controls in search of predictive biomarker combinations.

The large number of differentially expressed genes in MUTZ-3 cells stimulated with sensitizing chemicals vs. non-sensitizing controls revealed that MUTZ-3 indeed had a capacity to differentiate between these two groups. Efforts have previously been done to create assays based on genome analysis in various cell systems, such as e.g. CD34^+^-progenitor cells-derived DCs [22-24]. While such assays might provide in vivo like environments, primary cells are not well suited for a high-throughput format considering both donor-dependent variations as well as ethical aspect of such cell sources. Furthermore, previous efforts within in vitro assay development for sensitization that rely on full genome analysis have used a limited set of testing compounds.

The present study utilized in all 40 compounds and efforts were made to divide these compounds into two subsets, for training and testing respectively. While these experiments have resulted in successful predictions (data not shown), it is our experience that sensitizing compounds differ greatly in their induced gene expression profile, as can be seen in Figure 3D. In this perspective, we strived to include as many training compounds as possible when identifying our Prediction Signature, and did not exclude any compounds for validation. Instead, we validated the method by which the Prediction Signature was identified, by subdividing the samples into training and test sets at random, using unseen data for validation, to avoid overfitting. At present, the Prediction Signature consists of 200 transcripts, based on Figure 4A. Continuing the elimination process beyond 200 transcripts causes loss of information, as seen by the rise of KLD. Experiments have shown that correct classifications are possible even with further reduced signatures, down to 11 genes (data not shown). A reduction of signature size could be assessed in conjunction with validation of the assay, using untested positive and negative compounds in a new test set. By reducing the signature size at this point, the risk of biasing the signature towards this data set increases, making it harder to correctly classify unknown samples. Additional test compounds will also serve to assess the frequency of extreme transcriptional profile outliers, such as Oxazolone and Cinnamic aldehyde, which had to be removed from the analysis performed in this study. A number of reasons may be attributed to the fact that these compounds were not compatible with the assay, such as solubility in the cell media or extreme toxic effects. In those cases, other in vitro alternatives may complement this assay, so that the safety assessment of chemicals for sensitization includes a battery of in vitro assays. Naturally, an additional data set with blinded compounds is essential to validate whether the assay truly performs as estimated by the random subdivisions into training and test sets.

Of note, our Prediction Signature is able to predict the potency of sensitizing compounds, as defined by the LLNA (Figure 4C). However, the potency predicted by LLNA and that of our classifier do not match for all samples. Notably, the moderate sensitizer 2-hydroxyethyl acrylate showed resemblance to strong and extreme sensitizers with respect to gene expression profile. Similarly, the moderate sensitizers ethylendiamine, hexylcinnamic aldehyde, and glyoxal grouped together with weak sensitizers. These findings support the fact that sensitizing potency, as defined, may need revising.

By studying the identity of the transcripts and their involvement in intracellular signaling pathways, we were also able to confirm the biological relevance of the Prediction Signature. Using IPA, we found that the most highly populated pathways were nuclear factor-erythroid 2-related factor 2 (NRF2) mediated oxidative response, xenobiotic metabolism signaling, protein ubiquitination pathway, LPS/IL-1 mediated inhibition of Retinoic X receptor (RXR) function, aryl hydrocarbon receptor (AHR) signaling, and protein kinase A (PKA) signaling. These pathways are all known to take part in reactions provoked by xenobiotics, and several were associated with oxidative stress. Furthermore, Toll-like receptor (TLR) signaling is among the top pathways found in IPA. Recent studies on assay development for prediction of sensitization in vitro have to a large extent focused on how danger signals are provided to antigen-presenting cells, inducing pro-inflammatory cytokines and chemokines, as well as co-stimulatory molecules needed for a specific T-cell response. We hypothesize that these signals are provided through the innate immune responses, in analogy with infections, as reviewed in [25].

The primary pathways found in this study involved NRF2 signaling. This is a pathway activated by Reactive Oxygen Species (ROS), and is a defense mechanism to xenobiotics and response to cellular stress. In the resting cell, NRF2 is bound by kelch-like ECH-associated protein 1 (KEAP1) and located in the cytosol. In the response to ROS activity, KEAP1 is targeted for ubiquitination and protesomal degradation, resulting in the translocation of NRF2 to the nucleus, where it activates transcription of genes containing anti-oxidant response elements (ARE) in their promoter region [26]. The functions of genes transcribed by NRF2 association to ARE include regulation of inflammation, migration of DC and anti-oxidant defense enzymes, such as NADPH quinone oxidoreductase 1 (NQO1) and glutathione S-transferases (GST) [27,28], genes found in the Prediction Signature. Furthermore, the NRF2/KEAP1/ARE pathway has previously been described as activated in response to skin sensitizers, inducing maturation of dendritic cells [29].

Similarly, AHR is a transcription factor in the cytosol that is activated by binding to ligands, which includes a wide range of xenobiotic chemicals, such as halogenated aromatic hydrocarbons, polyphenols and a number of pharmaceuticals [30]. In the absence of a ligand, AHR is bound by a complex of chaperon proteins, keeping it in the cytosol. Upon ligand binding, AHR is translocated to the nucleus, where it dimerizes with aryl hydrocarbon receptor nuclear translocator (ARNT) [30]. The ARNT/AHR heterodimer then binds to xenobiotic response elements (XRE) in promoter regions of target genes. The typical target genes for XRE include enzymes for drug metabolism, such as the cytochrome P450 (CYP) superfamily, as well as cytoprotective enzymes mediating defense against oxidative stress, such as NQO1 [31]. Interestingly, while NQO1 is under control of both NRF2 and AHR, with both ARE and XRE in the promoter region, it has also been shown that AHR is among the target genes for the activated NRF2 pathway and vice versa [32]. Thus, a battery of protective enzymes are induced in response to a variety of xenobiotics, possibly through a number of signaling pathways, ultimately leading to the maturation of dendritic cells, as also indicated by the present data. The protein ubiquitination pathway is involved in degradation of short-lived or regulatory proteins involved in many cellular processes, such as the cell cycle, cell proliferation, apoptosis, DNA repair, transcription regulation, cell surface receptors and ion channels regulation, and antigen presentation. Of note, both NRF2 and AHR are in the resting cell bound by proteins that are targeted for ubiquitination upon ligand binding.

RXR is a nuclear receptor, with retinoic acid as the most prominent natural ligand [33]. It has previously been described as important for xenobiotics recognition and glutathione homeostasis, with cytoprotective enzymes as target genes [34,35].

TLR signaling is known to play a major role in dendritic cell maturation, as they activate transcription of a number of pro-inflammatory cytokines, chemokine-receptors for homing to lymph nodes and co-stimulatory molecules [36-38]. While TLR6 and TLR9 are present in our Prediction Signature, others have reported TLR4 as a crucial mediator of contact allergy to nickel [39]. As these receptors all signal through nuclear factor kappa-light-chain-enhancer of activated B cells (NF-κB), it is not surprising that different compounds activate different receptors, considering the chemical diversity of skin sensitizers, as discussed above.

Lastly, PKA signaling is a vastly versatile pathway activated by numerous stimuli, and, to the best of knowledge, this pathway has not previously been reported in association with skin sensitization. However, individual species of CYPs are known to be phosphorylated by PKA, in response to elevated levels of cyclic adenosine monophosphate (cAMP), triggered by xenobiotics. In addition, cAMP levels influence the nuclear translocation of AHR, connecting these two pathways and their impact on CYP activity [40].

## Conclusion

In this paper, we have demonstrated the predictive power of a genomic biomarker signature, which correctly classifies sensitizers and non-sensitizers. The biomarker signature was derived from the human DC-like cell line MUTZ-3, which was challenged with a panel of 40 reference chemical compounds. The biomarker genes were shown to be biologically relevant, as demonstrated by their involvement in cytoprotective mechanisms and pathways triggered by xenobiotic substances, supporting their relevance as predictor genes for skin sensitization. The findings reported in this paper might impact the development of in vitro assays for assessment of skin sensitization, which is crucial in order to replace the animal models currently in use.

## Methods

### Chemicals

A panel of 40 chemical compounds, consisting of 20 sensitizers and 20 non-sensitizers were used for cell stimulations. The sensitizers were 2,4-dinitrochlorobenzene, cinnamaldehyde, resorcinol, oxazolone, glyoxal, 2-mercaptobenzothiazole, eugenol, isoeugenol, cinnamic alcohol, *p*-phenylendiamine, formaldehyde, ethylendiamine, 2-hydroxyethyl acrylate, hexylcinnamic aldehyde, potassium dichromate, penicillin G, kathon CG (MCI/MI), 2-aminophenol, geraniol and 2-nitro-1,4-phenylendiamine. The non-sensitizers were sodium dodecyl sulphate, salicylic acid, phenol, glycerol, lactic acid, chlorobenzene, *p*-hydrobenzoic acid, benzaldehyde, diethyl phtalate, octanoic acid, zinc sulphate, 4-aminobenzoic acid, methyl salicylate, ethyl vanillin, isopropanol, dimethyl formamide, 1-butanol, potassium permanganate, propylene glycol and tween 80 (Table 3). All chemicals were from Sigma-Aldrich, St. Louis, MO, USA. Compounds were dissolved in either dimethyl sulfoxide (DMSO) or distilled water. Prior to stimulations, the cytotoxicity of all compounds was monitored, using propidium iodide (PI) (BD Biosciences, San Diego, CA) using protocol provided by the manufacturer. The relative viability of stimulated cells was calculated as

**Table 3:** List of reference chemicals used in assay development

Compound	Abbreviation	Potency	LLNA	HMT^1^	HPTA^1^
***Sensitizers***					
2,4-Dinitrochlorobenzene	DNCB	Extreme [15]	+ [15]		
Oxazolone	OXA	Extreme [15]	+ [15]		
Potassium dichromate	PD	Extreme [14]	+ [14]	+	+
Kathon CG (MC/MCI)	KCG	Extreme [14,45]	+ [14,46]		
Formaldehyde	FA	Strong [15]	+ [15]	+	+
2-Aminophenol	2AP	Strong [46]	+ [47]		
2-nitro-1,4-Phenylendiamine	NPDA	Strong [46]	+ [47]		
p-Phenylendiamine	PPD	Strong [47]	+ [48]	+	+
Hexylcinnamic aldehyde	HCA	Moderate [15]	+ [15]		
2-Hydroxyethyl acrylate	2HA	Moderate [46]	+ [47]		+
2-Mercaptobenzothiazole	MBT	Moderate [46]	+ [47]	+	+
Glyoxal	GO	Moderate [46]	+ [47]	+	
Cinnamaldehyde	CALD	Moderate [47]	+ [48]	+	+
Isoeugenol	IEU	Moderate [47]	+ [48]		+
Ethylendiamine	EDA	Moderate [14]	+ [14]		
Resorcinol	RC	Moderate [48]	+ [49]	-	+
Cinnamic alcohol	CALC	Weak [46]	+ [48]		
Eugenol	EU	Weak [47]	+ [48]		+
Penicillin G	PEN G	Weak [47]	+ [48]	+	
Geraniol	GER	Weak [14]	+ [14]	-	+
***Non-sensitizers***					
1-Butanol	BUT		- [50]		
4-Aminobenzoic acid	PABA		- [51]	-	+
Benzaldehyde	BA		- [52]		
Chlorobenzene	CB		- [14]		
Diethyl phthalate	DP		- [48]		
Dimethyl formamide	DF		- [46]		
Ethyl vanillin	EV		- [52]		
Glycerol	GLY		- [48]		
Isopropanol	IP		- [48]		
Lactic acid	LA		- [14]		
Methyl salicylate	MS		- [14]	-	
Octanoic acid	OA		- [53]		
Propylene glycol	PG		- [51]		
Phenol	PHE		- [53]	-	
p-Hydroxybenzoic acid	HBA		- [54]		
Potassium permanganate	PP		-		
Salicylic acid	SA		- [14]	-	
Sodium dodecyl sulphate	SDS		+^2 ^[14,53]	-	
Tween 80	T80		- [20]		+
Zinc sulphate	ZS		+^2 ^[55]		

List of sensitizers and non-sensitizers used in assay development. 1) HMT, Human Maximization Test; HPTA, Human Patch Test Allergen. Information is derived from [17]. 2) False positives in LLNA.

For toxic compounds, the concentration yielding 90% relative viability (Rv90) was used. For non-toxic compounds, a concentration of 500 μM was used. For non-toxic compounds that were insoluble at 500 μM in medium, the highest soluble concentration was used. For compounds dissolved in DMSO, the final concentration of DMSO in each well was 0.1%. The vehicle and concentrations used for each compound are listed in Table 4.

**Table 4:** Concentrations and vehicles used for each reference chemical

Compound	Abbreviation	Vehicle	Max solubility (μM)	Rv90 (μM)	Concentration in culture (μM)
***Sensitizers***					
2,4-Dinitrochlorobenzene	DNCB	DMSO	-	4	4
Oxazolone	OXA	DMSO	250	-	250
Potassium dichromate	PD	Water	51.02	1.5	1.5
Kathon CG (MC/MCI)^1^	KCG	Water	-	0.0035%	0.0035%
Formaldehyde	FA	Water	-	80	80
2-Aminophenol	2AP	DMSO	-	100	100
2-nitro-1,4-Phenylendiamine	NPDA	DMSO	-	300	300
p-Phenylendiamine	PPD	DMSO	566	75	75
Hexylcinnamic aldehyde	HCA	DMSO	32.34	-	32.24
2-Hydroxyethyl acrylate	2HA	Water	-	100	100
2-Mercaptobenzothiazole	MBT	DMSO	250	-	250
Glyoxal	GO	Water	-	300	300
Cinnamaldehyde	CALD	Water	-	120	120
Isoeugenol	IEU	DMSO	641	300	300
Ethylendiamine	EDA	Water	-	-	500
Resorcinol	RC	Water	-	-	500
Cinnamic alcohol	CALC	DMSO	500	-	500
Eugenol	EU	DMSO	649	300	300
Penicillin G	PEN G	Water	-	-	500
Geraniol	GER	DMSO	-	-	500
***Non-sensitizers***					
1-Butanol	BUT	DMSO	-	-	500
4-Aminobenzoic acid	PABA	DMSO	-	-	500
Benzaldehyde	BA	DMSO	250	-	250
Chlorobenzene	CB	DMSO	98	-	98
Diethyl phthalate	DP	DMSO	50	-	50
Dimethyl formamide	DF	Water	-	-	500
Ethyl vanillin	EV	DMSO	-	-	500
Glycerol	GLY	Water	-	-	500
Isopropanol	IP	Water	-	-	500
Lactic acid	LA	Water	-	-	500
Methyl salicylate	MS	DMSO	-	-	500
Octanoic acid	OA	DMSO	504	-	500
Propylene glycol	PG	Water	-	-	500
Phenol	PHE	Water	-	-	500
p-Hydroxybenzoic acid	HBA	DMSO	250	-	250
Potassium permanganate	PP	Water	38	-	38
Salicylic acid	SA	DMSO	-	-	500
Sodium dodecyl sulphate	SDS	Water	-	200	200
Tween 80	T80	DMSO	-	-	500
Zinc sulphate	ZS	Water	126	-	126

List of concentrations and vehicles used for each testing compound. 1) Kathon CG is a mixture of the compounds MC and MCI. The concentration of this mixture is given in %.

### Chemical exposure of the cells

The human myeloid leukemia-derived cell line MUTZ-3 (DSMZ, Braunschweig, Germany) was maintained in α-MEM (Thermo Scientific Hyclone, Logan, UT) supplemented with 20% (volume/volume) fetal calf serum (Invitrogen, Carlsbad, CA) and 40 ng/ml rhGM-CSF (Bayer HealthCare Pharmaceuticals, Seattle, WA), as described [10]. Cultures were maintained at 200.000 cells/ml during expansion, with a media change every 3-4 days. No differentiating steps were performed. Instead, the proliferating progenitor MUTZ-3 was used for stimulations, as delivered by the supplier. Prior to each experiment, the cells were immunophenotyped using flow cytometry as a quality control. Cells were seeded in 6-well plates at 200.000 cells/ml. Stock solutions of each compound were prepared in either DMSO or distilled water, and were subsequently diluted so the in-well concentrations corresponded to the Rv90 value, and in-well concentrations of DMSO were 0.1%. Cells were incubated for 24 h at 37°C and 5% CO_2_. Thereafter, cells were harvested and analyzed by flow cytometry. In parallel, harvested cells were lysed in TRIzol reagent (Invitrogen) and stored at -20°C until RNA extraction. Stimulations with chemicals were performed in three individual experiments, so that triplicates samples were obtained.

### Phenotypic analysis with flow cytometry

All cell surface staining and washing steps were performed in PBS containing 1% BSA (w/v). Cells were incubated with specific mouse mAbs for 15 min at 4°C. The following mAbs were used for flow cytometry: FITC-conjugated CD1a (DakoCytomation, Glostrup, Denmark), CD34, CD86, and HLA-DR (BD Biosciences), PE-conjugated CD14 (DakoCytomation), CD54 and CD80 (BD Biosciences). Mouse IgG1, conjugated to FITC or PE were used as isotype controls (BD Biosciences) and PI was used to assess cell viability. FACSDiva software was used for data acquisition with FACSCanto II instrument (BD Bioscience). 10,000 events were acquired and gates were set based on light scatter properties to exclude debris and nonviable cells. Further data analysis was performed using FCS Express V3 (De Novo Software, Los Angeles, CA).

### Preparation of cRNA and gene chip hybridization

RNA isolation and gene chip hybridization was performed as described [41]. Briefly, RNA from unstimulated and chemical-stimulated MUTZ-3 cells, from triplicate experiments, were extracted and analyzed. The preparation of labeled sense DNA was performed according to Affymetrix GeneChip™ Whole Transcript (WT) Sense Target Labeling Assay (100 ng Total RNA Labeling Protocol) using the recommended kits and controls (Affymetrix, Santa Clara, CA). Hybridization, washing and scanning of the Human Gene 1.0 ST Arrays were performed according to the manufacturer's protocol (Affymetrix). The microarray data have been deposited in the Array Express database http://www.ebi.ac.uk/arrayexpress/ with accession number E-MTAB-670.

### Microarray data analysis and statistical methods

The microarray data were normalized and quality checked with the RMA algorithm, using Affymetrix Expression Console (Affymetrix). Genes that were significantly regulated when comparing sensitizers with non-sensitizers were identified using one-way ANOVA, with false discovery rate (FDR) as a correction for multiple hypothesis testing. In order to reduce the large number of identified significant genes, we applied an algorithm developed in-house for Backward Elimination of analytes [42]. With this method, we train and test a Support Vector Machine (SVM) model [12] with leave-one out cross-validation, with one analyte left out. This process is iterated until each analyte has been left out once. For each iterative step, a Kullback-Leibler divergence (KLD) is recorded, yielding *N *KLDs, where *N *is the number of analytes. The analyte that was left out when the smallest KLD was observed is considered to provide the least information in the data set. Thus, this analyte is eliminated and the iterations proceed, this time with *N*-1 analytes. In this manner, the analytes are eliminated one by one until a panel of markers remain that have been selected based on the ability of each analyte to contribute with orthogonal information for the discrimination of skin sensitizers vs. non-sensitizers. The selected biomarker profile of 200 transcripts were designated the "Prediction Signature". The scripts for Backwards Elimination and Support Vector Machines were programmed for R [43], with the additional package e1071 [44]. ANOVA analyses and visualization of results with Principal Component Analysis were performed in Qlucore Omics Explorer 2.1 (Qlucore AB, Lund, Sweden). Hierarchical clustering for the heatmap was performed in R.

### Interrogation of the method for identification of the Prediction Signature

The data set was divided into a training set and a test set, consisting of 70% and 30%, of the chemical compounds, respectively. The division was performed randomly, while maintaining the proportions of sensitizers and non-sensitizers in each subset at the same ratio as in the complete data set. A biomarker signature was identified in the training set, using ANOVA filtering and Backward Elimination, as described above. This test signature was used to train an SVM, using the training set, which was thereafter applied to predict the samples of the test set. The process was repeated 20 times and the distribution of the area under the Receiver Operating Characteristic (ROC) curve [45] was used as a measurement of the performance of the model.

### Assessment of biological functions of Prediction Signature using pathway analysis

In order to investigate the biological functions the gene profile of the 200 genes derived from the Backward Elimination was analyzed, using the Ingenuity Pathway Analysis software, IPA, (Ingenuity Systems, Inc. Mountain View, USA). The gene profile was analyzed using the 'Build' and 'Path Explorer' functions to build an interactome of the core genes from the Prediction Signature together with connecting molecules, as suggested by IPA. The molecules of the signature were connected using the shortest known paths. In this process only human data from primary cells, cell lines and epidermal tissue was used. Public identifiers were used to map genes in IPA. All molecules except for endogenous and chemical drugs were allowed in the network and all kinds of connections were allowed. Known 'Functions' and 'Canonical Pathways' from IPA were mapped to the signature using the 'Overlay' function. The most densely populated pathways and functions were reported. All were significant, using the built in IPA statistical measures (p-values for functions and -log(p-values) for pathways).

## Abbreviations

ACD: atopic contact dermatitis; AML: acute myeloid leukemia cell; APC: Antigen Presenting Cell; DC: Dendritic Cell; GM-CSF: Granulocyte macrophage colony-stimulating factor; GPMT: Guinea pig maximization test; HMT: Human Maximation Test; HPTA: Human Patch Test Allergen; IL: Interleukin; LLNA: Local Lymph Node Assay; PCA: Principal Component Analysis.

## Competing interests

The authors have applied for a patent related to the content of this article.

## Authors' contributions

ML and CB designed the study strategy. HJ and ML set up and optimized the cell-based assay. HJ performed the cellular stimulations with chemicals. HJ and ML wrote the manuscript. AA and HJ analyzed the microarray data and prepared the figures. All authors revised and approved the manuscript.
